# Supplementary figures and images for: Canonical Response Parameterization: Quantifying the structure of responses to single-pulse intracranial electrical brain stimulation
Source: PLoS Comput Biol. 2023 May 25;19(5):e1011105. doi: 10.1371/journal.pcbi.1011105 (PMC10246848; doi:10.1371/journal.pcbi.1011105)

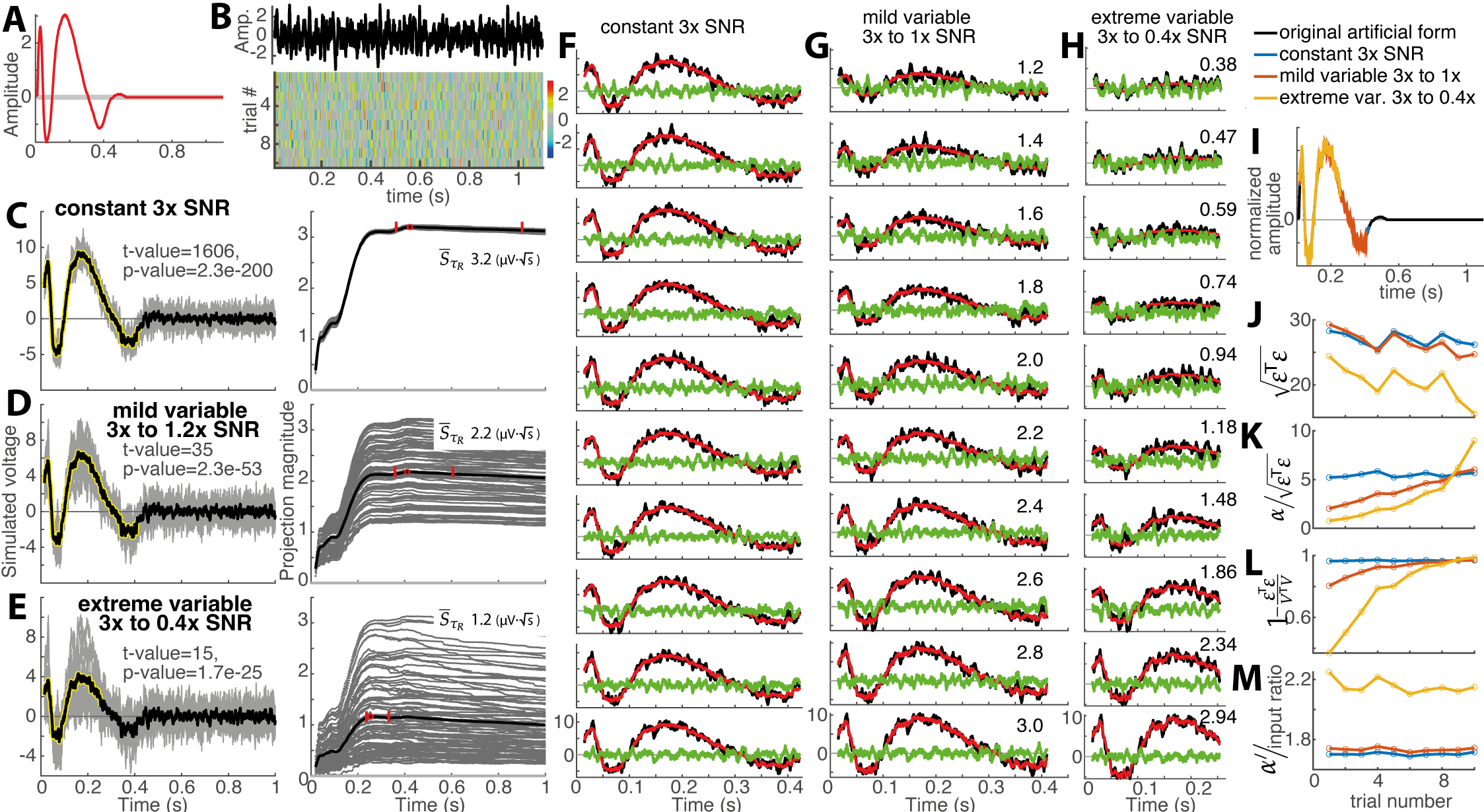

Supplement: S1 Fig — A. An artificial signal trace, normalized to variance of 1. B. 10 trials of brown-noise (i.e. random walk) timeseries, with each normalized to z-score of 1. Brown noise generated by cumulative sum of random data on -0.5 to 0.5 interval and subtracting off of running mean. C. Response duration (left) and timecourse of projection weights (right) extracted from synthetic traces with noise traces from (B) added to signal trace at ratio of 3-to-1. D. Response duration (left) and timecourse of projection weights (right) extracted from synthetic traces with noise traces from (B) added to signal trace at “mild” variable ratios of 1.2 to 3.0 in 0.2 intervals. E. Response duration (left) and timecourse of projection weights (right) extracted from synthetic traces with noise traces from (B) added to signal trace at “extreme” variable ratios of {0.38; 0.47; 0.59; 0.74; 0.94; 1.18; 1.48; 1.86; 2.34; 2.94}. F. Parameterization of the artificial evoked responses of constant signal-to-noise ratio of 3-to-1 (from (C)). G. Parameterization of the artificial evoked responses of the “mild” variable signal-to-noise ratios of 1.2 to 3.0 in 0.2 intervals (from (D)). H. Parameterization of the artificial evoked responses of the “extreme” variable ratios of {0.38; 0.47; 0.59; 0.74; 0.94; 1.18; 1.48; 1.86; 2.34; 2.94} (from (E)). I. Extracted C(t) for different noise levels overlaid on top of original artificial form. J. Single-trial noise residuals for different noise levels. K. Single-trial α′ to noise residual (SNR) for different noise levels. L. Single-trial explained variance for different noise levels. M. Single-trial ratio of coefficient α′ to input SNR for different noise levels. Differences between extreme variable traces (yellow) in (J) and (M) are due to shorter C(t). This shorter C(t) may be related to added correlated deviation toward zero by the brown noise statistics disproportionally contributing at higher noise levels. (TIF) [file pcbi.1011105.s001.tif]

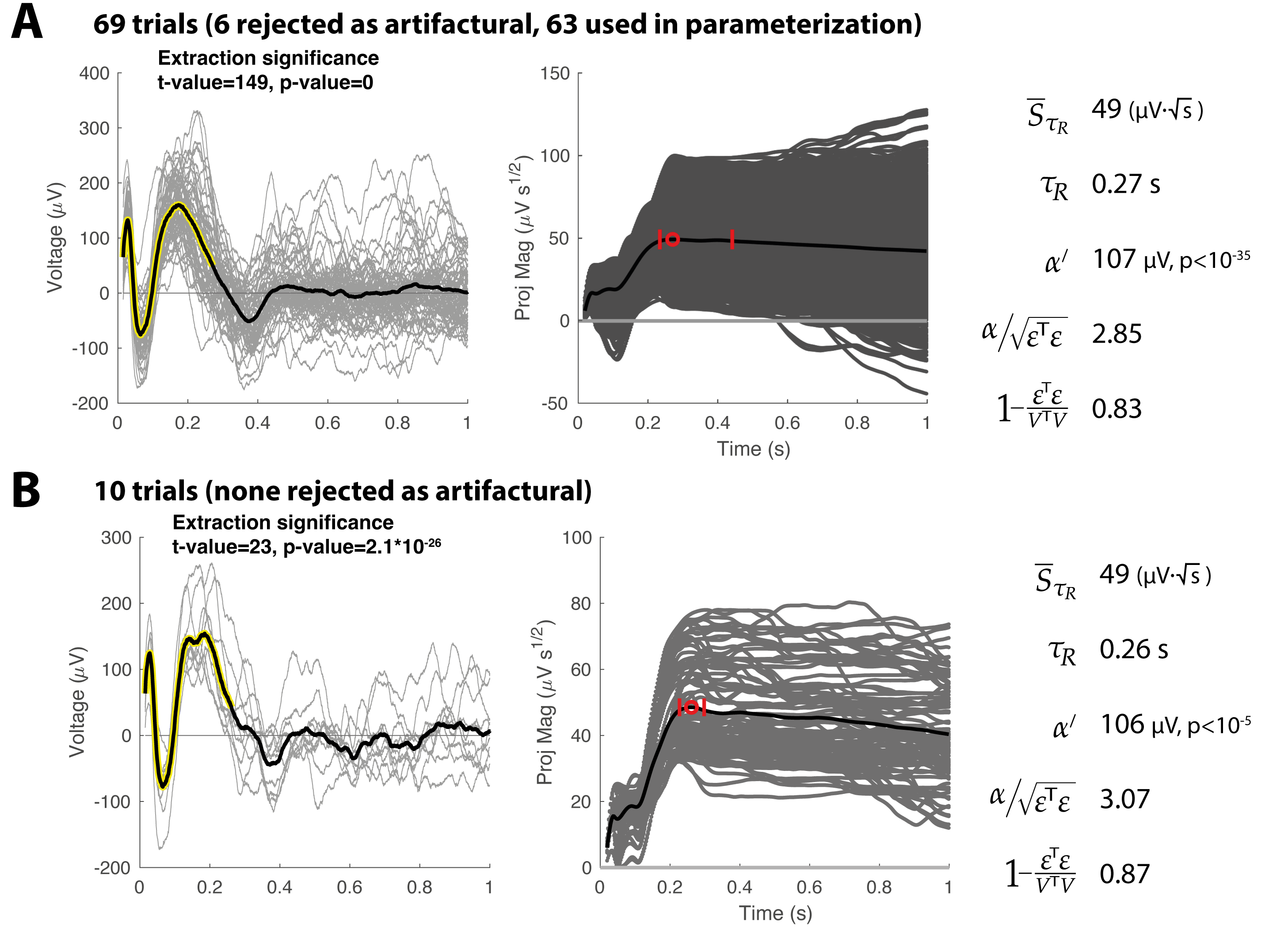

Supplement: S2 Fig — Measurement is from a dorsal insular contact in response to stimulation of white matter in the orbitofrontal cortex. A. Stimulation was performed 69 times. Artifact rejection was at a threshold of p <10−10, resulting in rejection of 6 trials. The extraction was robust with an associated t-value of 149. B. The first 10 trials from (A) were parameterized in an identical fashion. No trials were identified as artifactual, and the associated t-value for extraction was 23. Note that the response duration (τR), mean projection at response duration (S¯), and scaling coefficient (α′) were all nearly identical. However, the statistics of the parametrization were much more robust for 69 trials. The averaged explained variance and SNR were slightly higher for 10 trials (as might be expected). (TIF) [file pcbi.1011105.s002.tif]

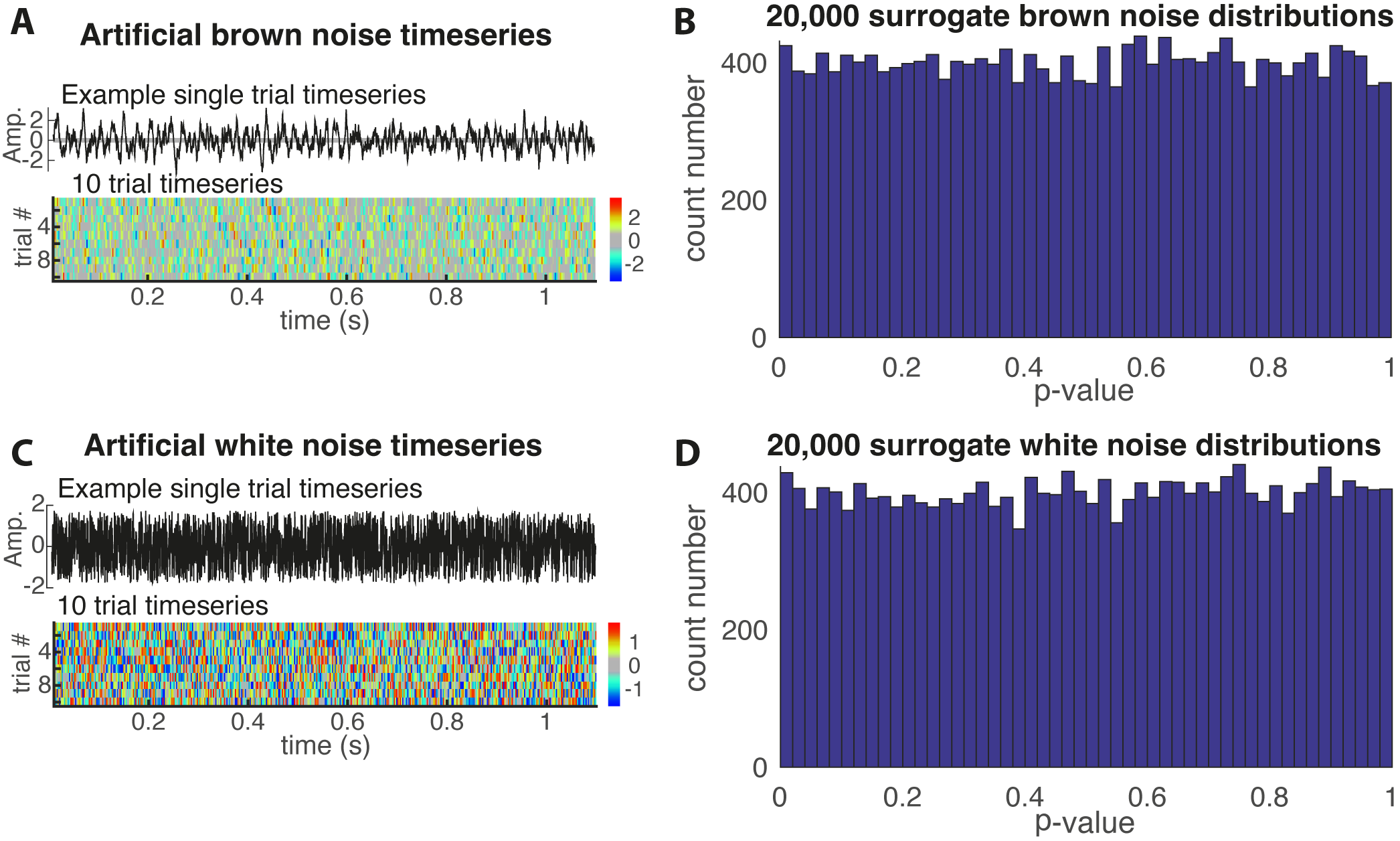

Supplement: S3 Fig — A. Top: An example of a single brown-noise (i.e. random walk) timecourse. Bottom: A 10-trial set of brown noise timecourses. B. A histogram of extraction significances from 20,000 surrogate sets of brown-noise timecourses. C. Top: An example of a single white-noise timecourse. Bottom: A 10-trial set of white-noise timecourses. D. A histogram of extraction significances from 20,000 surrogate sets of white-noise timecourses. Because histograms of p-values show a flat distribution over the 0-to-1 interval in (B) and (D), we may infer the statistical method is well calibrated for null models. (TIF) [file pcbi.1011105.s003.tif]

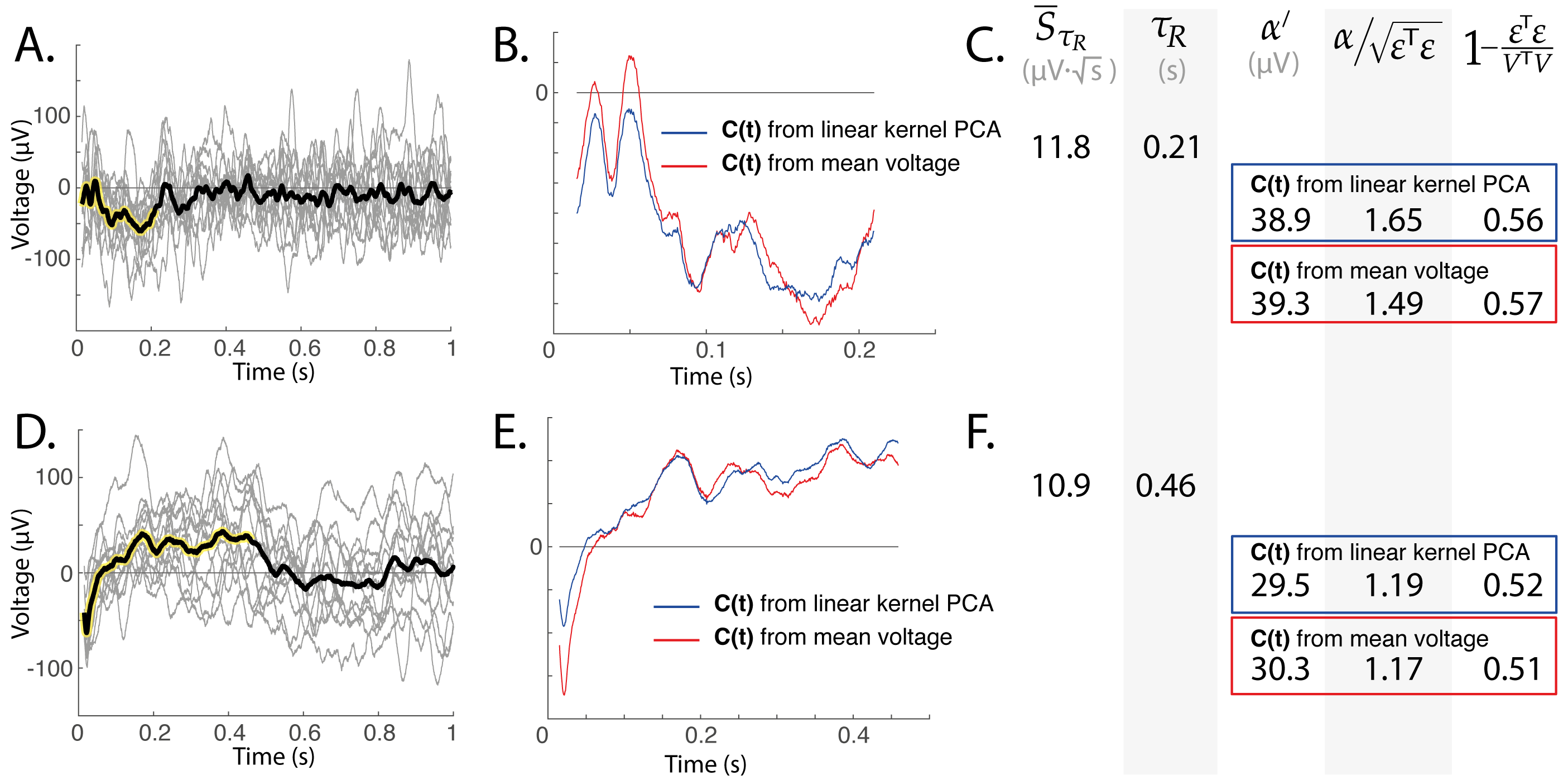

Supplement: S4 Fig — (A-C) are from the example in the middle row of Fig 5 and (D-F) are from Fig 8. A. The averaged voltage response, is shown with a black line, and the significant portion of the response is highlighted (i.e. up to τR). B. C(t) calculated from linear kernel PCA (blue) and from the simple mean (red). C. Parameterizations calculated from linear PCA vs mean voltage extractions. D-F. As in (A-C), for the example from Fig 8. (TIF) [file pcbi.1011105.s004.tif]
