# Supplementary material for: Canonical Response Parameterization: Quantifying the structure of responses to single-pulse intracranial electrical brain stimulation
Source: PLoS Comput Biol. 2023 May 25;19(5):e1011105. doi: 10.1371/journal.pcbi.1011105 (PMC10246848; doi:10.1371/journal.pcbi.1011105)
Supplement: Attachment — Submitted filename: kjm_CRP_reply_to_reviewers_R1.pdf [file pcbi.1011105.s005.pdf]

Mayo Clinic

Department of Neurosurgery
200 First Street SW
Rochester, Minnesota 55905

March 31, 2022

Dear Editors of *PLOS Computational Biology*,

This is a reply to reviewers for the manuscript "**Canonical Response Parameterization: Quantifying the structure of responses to single-pulse intracranial electrical brain stimulation**" (PCOMPBIOL-D-22-01199), by Miller, Müller, Ojeda Valencia, Huang, Gregg, Worrell, and Hermes.

We are very grateful for the thoughtful comments and suggestions from each of the reviewers. We feel that they have greatly improved the manuscript. All comments have been directly addressed, and the manuscript has been modified in response to each one. There were many modifications in the revision, but all were to specifically improve the manuscript in response to reviewer concerns.

I am personally grateful for your allowance of delay in the resubmission of the manuscript that arose from my personal medical issues, (which involved hospitalization and later surgery) beginning in Oct 2022.

Based upon the Biorxiv version uploaded at the time of initial submission, this technique has been adopted at several institutions. It features prominently in emerging work of ours as well as an accepted manuscript at *Nature* by Barbosa, et. al. ("An orexigenic subnetwork within the human hippocampus").

In this reply to reviewers document, gray text indicates commentary from the reviewer, **black** text indicates author response, and blue text indicates quotation from the manuscript. When required, LaTex format has been retained in responses.

Sincerely,

Kai J. Miller, Mayo Neurosurgery, on behalf of all authors

miller.kai@mayo.edu; Cellular phone (USA): +1 206 355 0244

Reviewer #1: The authors introduced a new method for quantifying the structure of the response to SPES. This method does not need a predetermined waveforms such as N1 or N2 by the examiner. Instead it employs semi-normalized dot-product projection to extract non-artifactual true response, determine the response duration, and then extract a characteristic shape for each recording contact using the Canonical Response Parameterization (CRP). They applied this methodology to the 2 patients with SEEG investigation, who underwent CCEP recording (10 stimulation per stimulus site). The concept is interesting in that we do not need a priori waveform as the previous SPES/CCEP literatures have defined, and that the method produces the canonical waveform and gets rid of the residual activities. The attempt is novel and interesting, but this new method needs some clarification for publication in the PLOS Computational Biology.

#1 Impact on artifact reduction
It is interesting to note that this method can find the outlier waveform (out of 10 waveforms obtained by 10 trials of stimulation). This is of clinical use when only the number of stimulation trials is small (10) as in this study. This may not be so effective for the stimulation trials over 50 trials. It would be nice if you have actual CCEP dataset of >50 trials and compare the effect of this method upon artifact reduction (or do some sort of simulation).

We thank the reviewer for the recommendation and have implemented it. As a general principle, techniques like these are more effective when there are more trials, rather than less trials. It is a particular feature of this method that it is robust even when there are only a few trials. In the intervening period, we were able to run an experiment where a single site was stimulated 69 times. Consider an example that illustrates an extraction performed on 69 trials, and on a subset of the first 10 trials in isolation.

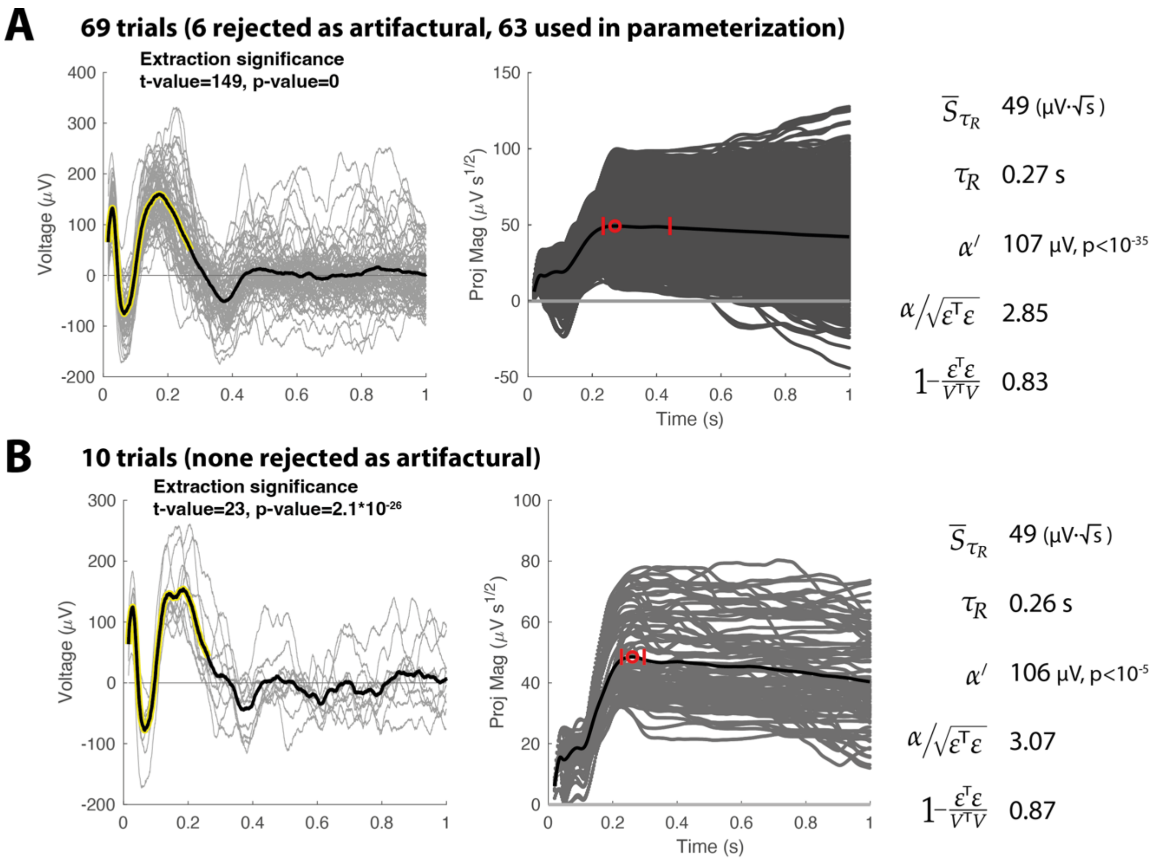

Figure R1: Example of parameterization with a large number of trials. Measurement is from a dorsal insular contact in response to stimulation of white matter in the orbitofrontal cortex. **(A)** Stimulation was performed 69 times. Artifact rejection was at a threshold of $p<10^{-10}$, resulting in rejection of 6 trials. The extraction was robust with an associated t-value of 149. **(B)** The first 10 trials from (A) were parameterized in an identical fashion. No trials were identified as artifactual, and the associated t-value for extraction was 23. Note that the response duration (τ_R), mean projection at response duration ($\bar{S}$), and scaling coefficient (α') were all nearly identical. However, the statistics of the parametrization were much more robust for 69 trials. The averaged explained variance and SNR were slightly higher for 10 trials (as might be expected).

We have included this figure as Supplemental Fig. S2, and added text referring readers to it in the subsection "Parameterization of single trials by canonical CRP shapes, $C(t)$, magnitude of the voltage response, α_{k}', and the residual, ε_k" of the Results and Discussion section: "While our illustrations consist of a low number of trials (10-15), the technique works easily and is associated with higher statistical significance when a much higher number of trials are obtained (Supplemental Fig. S2)."

It is also interesting to know how this method can get rid of the epileptic activity (usually larger than CCEP) that overrode (superimpose) on the CCEP response (which may occur only in some trials).

This CRP method can identify many trials that have epileptic activity that drowns out the CCEP response. However, this is not what the CRP method is designed for, and a technique designed to identify epileptic activity (rather than simply deviation from whatever the structure of the evoked response is) might do better at this. Consider the following example:

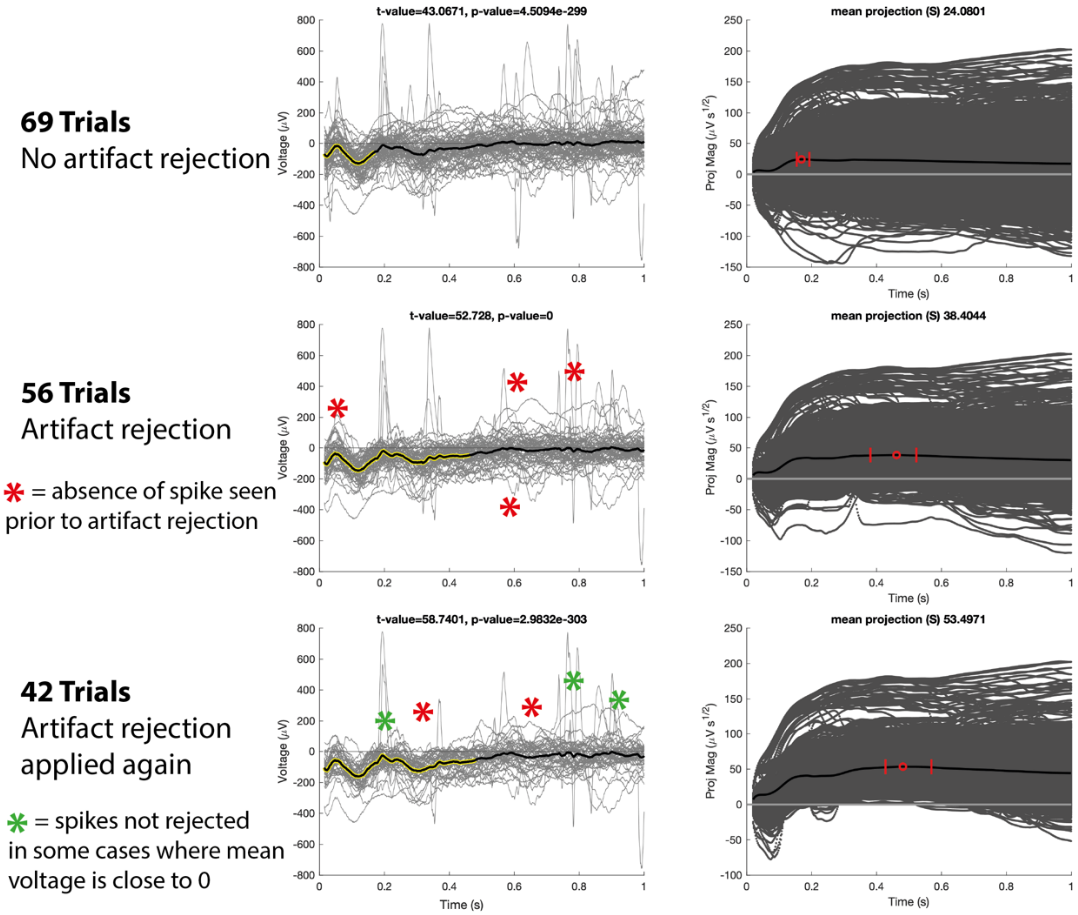

Figure R2: Evoked responses measured from the amygdala in a patient with very large epileptic spike discharges, while stimulating in the orbitofrontal white matter. The discharges are much larger in magnitude than the evoked responses. The trials with very large spike are mostly identified and rejected (red asterisks). However, some trials with spikes are not rejected when the spikes coincide with times when the mean voltage is centered around 0 (green asterisks). Note that rejection is for probability p<0.001 at each iteration.

#2 Significance of the method for clinical evaluation

As the authors pointed out typical N1/N2 waveforms are not always available especially in the case of SEEG recording. It would be nice to show more examples (CCEP findings form all the electrode contact (grey matter) for each stimulus site, and if possible, CCEP findings for more stimulus sites), since only a part of the CCEP waveforms is shown. It would be nice to see the effect of this method on the adjacent large CCEP response as well as the remote, relatively small CCEP responses. In addition, it is recommended to show these results together with their anatomical locations so that readers can compare the findings with the previous literatures.

Thank you for the recommendation. The purpose of this manuscript is to showcase a specific technique, including data for reproducing the illustrations. We are concerned that a wider application to a large patient cohort in this manuscript would be distracting from our primary purpose of illustrating this method. However, we have three manuscripts currently in review or published that have data which might be explored in this manner. Preprints of the manuscripts in submission are available on bioRxiv with links.

We have added text referring readers to these manuscripts in the subsection "Code and data availability" of the methods section: "…the reader interested in applying this technique with a wider set of example data than that included here can access further illustrative recordings released with our other emerging work by van Blooijs et. al. \cite{van2023developmental}, Ojeda Valencia et. al. \cite{ojeda2022Signatures} and Huang et. al. \cite{huang23154}."

From clinical point of view, if the authors can compare 1) mere averaging and 2) the results of their method (showing only C(t)) by showing their waveforms, then the readers from the clinical field can understand the utility of this method.

Thank you for this important suggestion. If one replaces the lines (script CRP_method.m, lines 93-94):

```
[E_tR,~]=kt_pca(V_tR); % Linear kernel trick PCA method to capture structure
C=E_tR(:,1); % 1st PC, canonical shape, C(t) from paper
```

with

```
C=mean(V_tR,2); C=C/norm(C); % mean shape
```

We can directly compare the extracted canonical form, and its parameterization, as seen in figure R3. This figure is now included as Supplemental Fig. S4

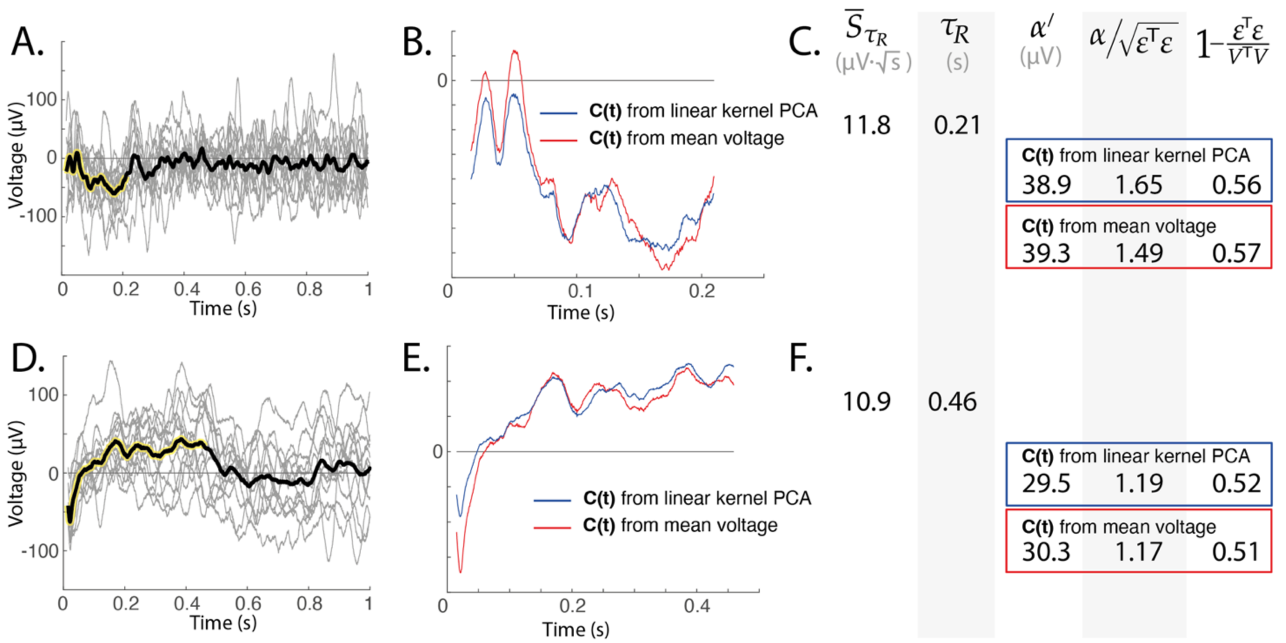

Figure R3: An illustration of calculating the canonical shape from linear kernel PCA or from the simple mean. (A-C) are from the example in the middle row of figure 5 and (D-F) are from figure 8. **(A)** The averaged voltage response, is shown with a black line, and the significant portion of the response is highlighted (i.e. up to τ_R). **(B)** C(t) calculated from linear kernel PCA (blue) and from the simple mean (red). **(C)** Parameterizations calculated from linear PCA vs mean voltage extractions. **(D-F)** As in (A-C), for the example from figure 8.

While we believe linear kernel PCA is better than the mean voltage, using the mean voltage does work reasonably well. We have included this figure as Supplemental Fig. S4. To address this in the text, the first item of the 'future directions' list at the end of the manuscript now reads:

"$C(t)$ could be chosen in different manners than we have, such as: using the simple average trace (i.e. $C(t) \longrightarrow \overline{V(t)}$ - we have anecdotally found that using the simple mean as the canonical form, rather than the PCA-based extraction, increases α', while reducing the SNR, as seen in supplemental Fig. S4);…"

We also feel that it might be good to de-emphasize the importance of using linear kernel PCA over the average, so we have removed the text "by using linear kernel PCA on V(t)" from previous line 252.

#3 Limitations
While the authors do acknowledge the timing issue of the CRP in limitations, the authors did not mention the small number of patients they studied (N = 2). The authors had better mention this since the application in the larger patient group may reveal some other important features of this new methodology.

As noted above, we are concerned that a wider application to a large patient cohort would be distracting from our primary purpose of illustrating this method, but we do agree that it would be helpful to be tested in a wider context.

In order to explicitly mention the limited number of patients, we have added text referring readers to these manuscripts in the subsection "Code and data availability" of the methods section: "Our illustration of this technique is limited to data from two patients. However, the reader interested in applying this technique with a wider set of example data than that included here can access further illustrative recordings released with our other emerging work by van Blooijs et. al. \cite{van2023developmental}, Ojeda Valencia et. al. \cite{ojeda2022Signatures} and Huang et. al. \cite{huang23154}."

Reviewer #2: Miller et al. present a new method for data-driven characterization of single-trial electrophysiological responses to neural stimulation. This method is developed in the context of "cortico-cortical evoked potentials" (CCEPs) in the human brain. These CCEPs are shifts in the (usually bulk) electrical field at site X induced by (usually bulk) electrical stimulation at site Y. In order to understand how electrical drive at one site (X) affects the response at another site (Y), a conventional approach has been to characterize CCEPs (evoked at site Y) via the timing and of their "peaks", especially the so-called N1/N2 maxima of the mean stimulation-evoked potential, which occur around 100 and 200ms post-stimulus. However, the authors argue that, for many pairs of cortical sites, the CCEPs do not exhibit (or are not well-characterized by) the N1/N2 components, so that a more general and flexible technique is required. The authors further argue that their approach (via linear kernel PCA) also allows the characterization of the duration of the evoked response, the computation of explained variance, as well as single trial response "magnitudes" and other features of the neural response.

This work is well illustrated and well argued, and it addresses a cornerstone methodological need for answering empirical questions around brain stimulation. This is valuable science, which certainly will make a contribution to the literature and to analytic practices of electrophysiological data.

The main drawback of this manuscript is that, although this method is clearly valuable and an advance on standard practice in human CCEP work, the manuscript does not provide the reader with (i) sufficient information about the conditions under which the method will work best and worst; and (ii) sufficiently detailed validation of the basic approach and statistical analysis methods. To be clear: my concern here is not that the method is invalid, but rather that its limits and domain of application are not clearly specified; this valuable work will be more immediately useful to other scientists if the limitations and assumptions are made clearer.

Main Points

* 1 * There appear to be several assumptions made in applying the linear kernel PCA method and using it to characterize the single-trial parameters and associates statistics. Please provide the reader with more information about these, and their justification. For example, does the model make assumptions about (i) the consistency of the timing of peaks and troughs across trials and (ii) the consistency of the magnitude of the signal waveform across trials?

Related to consistency of timing of peaks across trials: If the true underlying signal is a sharply ramping function (e.g. ramping from -1 to 1 in 20 milliseconds), will this not appear as a temporally "blurred" slowly ramping component (e.g. ramping from -1 to 1 in 80 milliseconds) if there is 50 ms of timing jitter from trial to trial? If the peak of a CCEP became later and later across stimulations (e.g. due to some kind of circuit adaptation effects), how would this affect the extracted C(t) curve.

Thank you - we perform the analysis as you suggest (see figure R4 below).

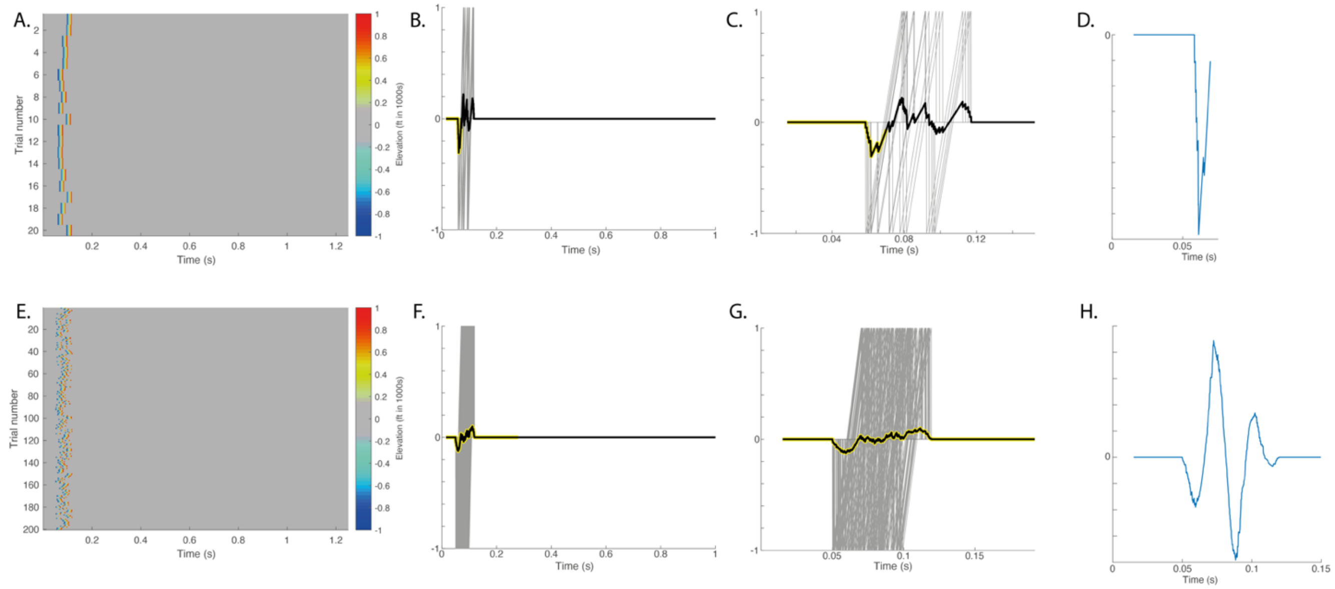

Figure R4: Illustration of a sharply ramping function (from -1 to 1 in 20 milliseconds), with 50ms of timing jitter from trial to trial. (A) 20 trials. **(B)** CRP extraction and parameterization of the 20 trials (α' significance p=0.001). **(C)** Zoom in of (B). **(D)** C(t) from the extraction of 20 trials. **(E)** 200 trials. **(F)** CRP extraction and parameterization of the 200 trials (α' significance p=0.24). **(G)** Zoom in of (F). **(H)** C(t) from the extraction of 200 trials.

Figure R4 shows that the extraction does not work well. When we perform the analysis with 20 trials, the extraction captures just the initial (negative) phase, with jagged blurring. It meets significance because the projections are strong enough for a few trials. When performed with 200 trials, the extraction captures 1) the shape of the ramp at the onset of the allowed period, and 2) the shape of the ramp at the offset of the allowed period (from the 50ms timing jitter). The shape of C(t) (Fig R4 H) for the 200 trial case does not look like the average response (in yellow in Fig R4 G). The reason for this is that the variation in timing is larger than the duration of either phase of the response.

The CRP approach, as noted, is not structured to deal with temporal variations. Future explorations might address this, allowing for jittered latency or temporal dilation. We feel that a full treatment of second-order expansions of this type that should be deferred to future since the central message (parameterization) would be clouded by content overload. However, we do note in a new bullet point in the "future exploration" list at the end of the manuscript:

"\item There is very little jitter in the response onsets of these CCEPs. In other contexts, such as ERP research, there is known variation in response onset, and expanded approaches will be needed in order to align trials temporally prior to parameterization."

Related to consistency of magnitude over trials: suppose that the neural circuits exhibit a form of adaptation in its response to stimulations, so that responses decay in magnitude over trials (i.e. the noise retains constant variance but the signal variance decreases over trials, while the signal waveform maintains the same shape). How would this affects the performance of the linear kernel PCA method?

Thank you - we perform the analysis as you suggest (see figure R5 and R6 below).

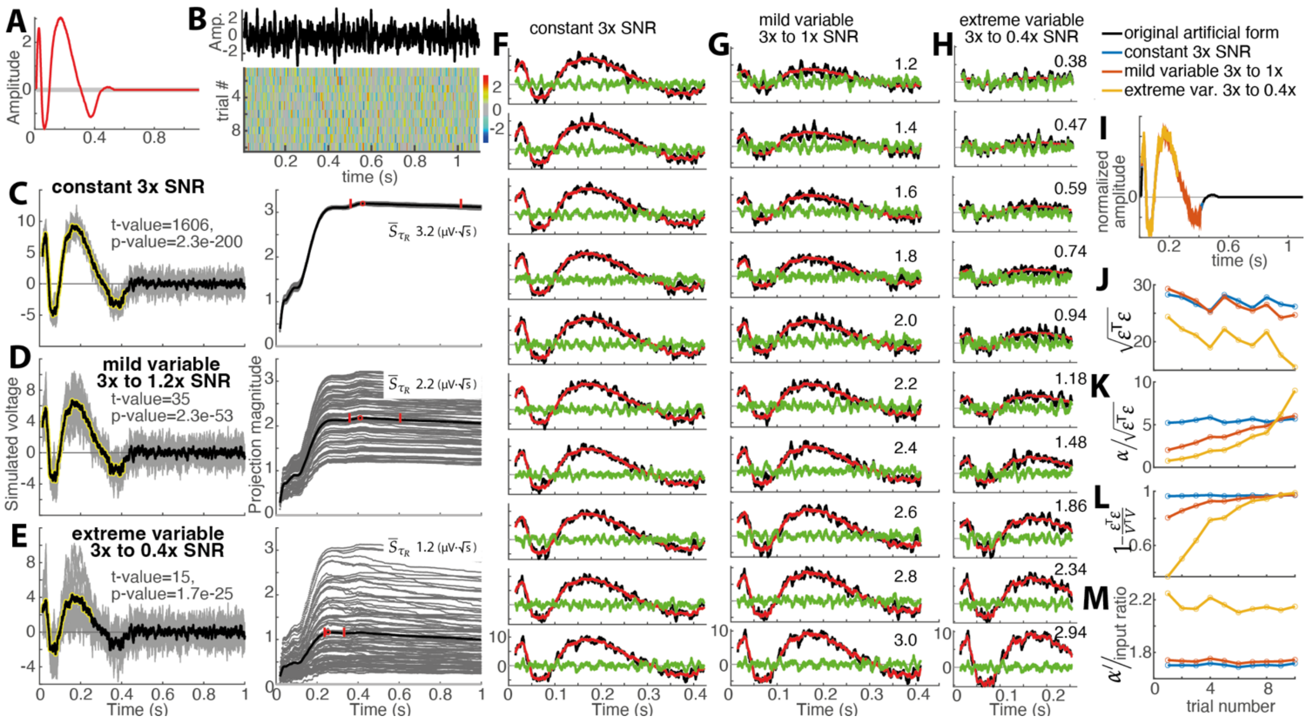

Figure R5: Artificially-generated evoked responses with variation in signal to noise. (A) An artificial signal trace, normalized to variance of 1. **(B)** 10 trials of brown-noise (i.e. random walk) timeseries, with each normalized to z-score of 1. Brown noise generated by cumulative sum of random data on -0.5 to 0.5 interval and subtracting off of running mean. **(C)** Response duration (left) and timecourse of projection weights (right) extracted from synthetic traces with noise traces from (B) added to signal trace at ratio of 3-to-1. **(D)** Response duration (left) and timecourse of projection weights (right) extracted from synthetic traces with noise traces from (B) added to signal trace at "mild" variable ratios of 1.2 to 3.0 in 0.2 intervals. **(E)** Response duration (left) and timecourse of projection weights (right) extracted from synthetic traces with noise traces from (B) added to signal trace at "extreme" variable ratios of {0.38; 0.47; 0.59; 0.74; 0.94; 1.18; 1.48; 1.86; 2.34; 2.94}. **(F)** Parameterization of the artificial evoked responses of constant signal-to-noise ratio of 3-to-1 (from (C)). **(G)** Parameterization of the artificial evoked responses of the "mild" variable signal-to-noise ratios of 1.2 to 3.0 in 0.2 intervals (from (D)). **(H)** Parameterization of the artificial evoked responses of the "extreme" variable ratios of {0.38; 0.47; 0.59; 0.74; 0.94; 1.18; 1.48; 1.86; 2.34; 2.94} (from (E)). **(I)** Extracted C(t) for different noise levels overlaid on top of original artificial form. **(J)** Single-trial noise residuals for different noise levels. **(K)** Single-trial α' to noise residual (SNR) for different noise levels. **(L)** Single-trial explained variance for different noise levels. **(M)** Single-trial ratio of coefficient α' to input SNR for different noise levels. Differences between extreme variable traces (yellow) in (J) and (M) are due to shorter C(t). This shorter C(t) may be related to added correlated deviation toward zero by the brown noise statistics disproportionally contributing at higher noise levels.

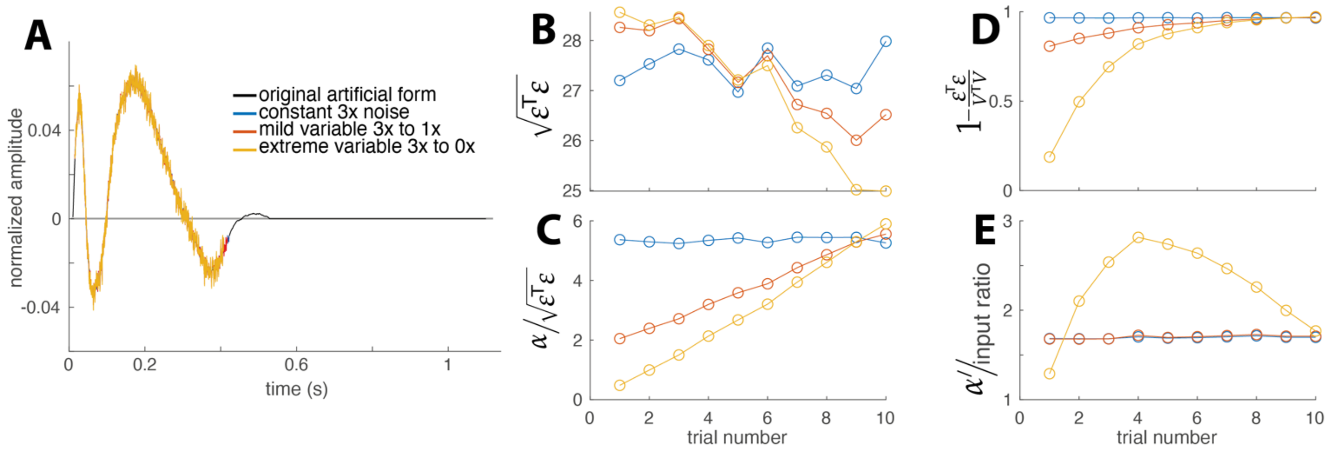

Figure R6: Artificially-generated evoked responses with variation in signal to noise, white noise added. (A) Extracted C(t) for different noise levels overlaid on top of original artificial form, with white noise added. Note that these differ from those extracted in figure R5(I) in that the C(t) are of similar length. **(B)** Single-trial noise residuals for different noise levels. **(C)** Single-trial α' to noise residual (SNR) for different noise levels. **(D)** Single-trial explained variance for different noise levels. **(E)** Single-trial ratio of coefficient α' to input SNR for different noise levels. Differences between extreme variable traces (yellow) in (J) and (M) are due to shorter C(t). The longer C(t) for the white noise is because deviations do not make larger deviations to the same degree that the correlation brown noise addition traces do.

As figure R5 and R6 demonstrate, the CRP technique is robust against diminishing signal in the presence of constant noise (whether it is white or brown noise). We have made this figure R5 our first supplemental figure, and have referred to this in the text in a new sentence in the first paragraph of the subsection "Parameterization of single trials by canonical CRP shapes, $C(t)$, magnitude of the voltage response, α_{k}', and the residual, ε_k" of the results section.

"Importantly, the CRP technique is quite robust and performs well with diminishing signal in the presence of constant noise (which we have found explicitly, using synthetically-generated responses - illustrated in Supplemental Fig. S1)."

* 2 * Are the statistical methods well-calibrated? When data are generated from a null model, do they indeed produce 5% false positive rates in simulated data, when the alpha value is set to 5%? Does the statistical approach control the false-positive rate (nominally 5%) at the level of each individual time point within a trial, or does the statistical approach control the false positive rate at the level of the whole trial, or the whole dataset?

We are grateful for the inquiry by the reviewer – it has led to a key improvement in our technique. In the first iteration of the analysis, we had included the full set of projections in the t-test. However, this creates artificial significance since a pair-wise interaction between two trials is counted twice. Once when the 1st trial is normalized & the 2nd trial is raw, and once in the inverse case.

To remedy this, only half of projections are now considered for p-value and t-value analysis. These are selected such that each trial is the normalized trial half of the time and raw half of the time, and pair-wise interactions are counted only once.

Having made this change, we then generated sets of surrogate brown- and white-noise timeseries, as illustrated in figure R7 below. Surrogate sets of 10 trials were generated, analyzed using the CRP approach, and tested for statistical significance. This process was repeated 20,000 times. Histograms of p-values from this show a flat distribution over the 0-to-1 interval, indicating that the statistical method is well calibrated for brown- and white-noise timeseries.

We have included figure R7 as Supplemental Fig. S3, and added text referring readers to it in the first paragraph of the subsection "Characterizing significance, anomalous trials, and artifact" of the Results and Discussion section: "The distribution of p-values obtained from many iterations of null data are evenly distributed on the 0-to-1 interval, indicating that the extracted significances are statistically appropriate (Supplemental Fig. S3)."

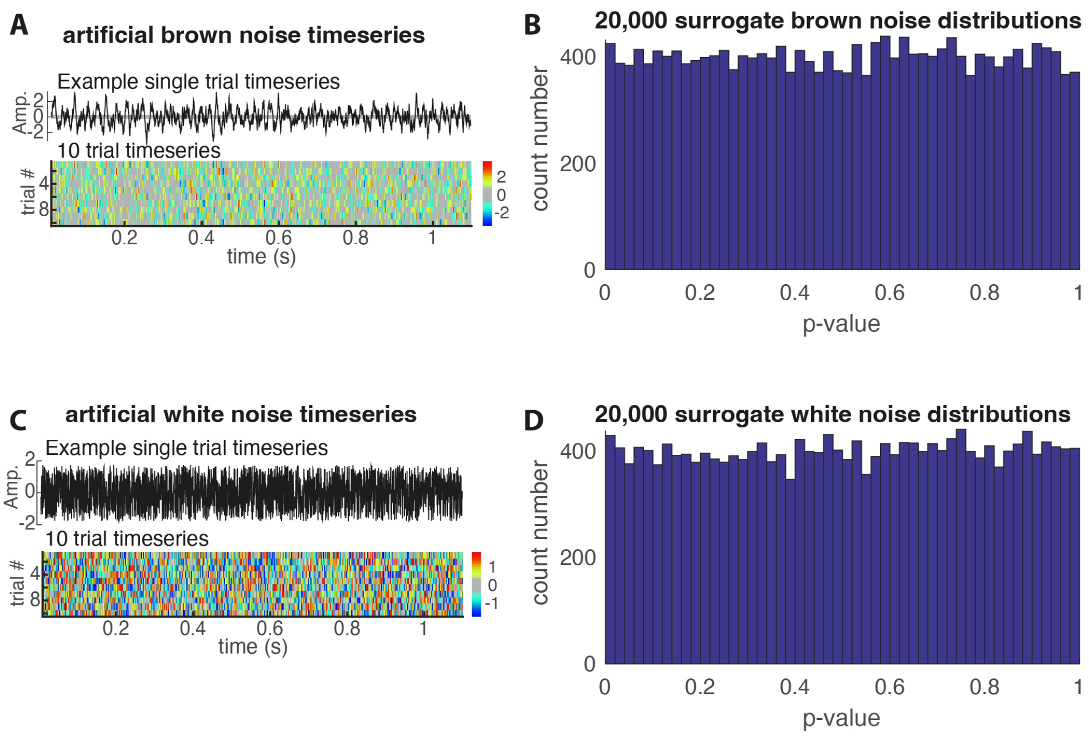

Figure R7: Generation of many artificially-generated sets of pure-noise data to validate statistics. (A) Top: An example of a single brown-noise (i.e. random walk) timecourse. Bottom: A 10-trial set of brown noise timecourses. **(B)** A histogram of extraction significances from 20,000 surrogate sets of brown-noise timecourses. **(C)** Top: An example of a single white-noise timecourse. Bottom: A 10-trial set of white-noise timecourses. **(D)** A histogram of extraction significances from 20,000 surrogate sets of white-noise timecourses. Because histograms of p-values show a flat distribution over the 0-to-1 interval in (B) and (D), we may infer the statistical method is well calibrated for null models.

We refer to this selection of half of the projections in Methods,

"Note that one cannot use the full distribution of S_{τ_R} - this creates artificial significance since a pair-wise interaction between two trials is counted twice. Once when the once when the first trial is normalized \& the second trial is raw, and once in the inverse case. Therefore, only half of projections are considered for p-value and t-value analysis. These are selected such that 1) each trial is the normalized trial half of the time and raw half of the time, and 2) pair-wise interactions are counted only once. Sub-distributions of S_{τ_R} corresponding to the projection magnitudes involving single trials can be used to identify the $``$most anomalous and most normal" single trials - (with comparison against the same selection of half of the projection magnitudes)."

It is also addressed in the subsection "Characterizing significance, anomalous trials, and artifact" of the Results:

"It should be noted that there is some relationship between conjugate cross-projection magnitudes $\sum_{t} \tilde{V}_{k}(t)V_{l}(t)$ and $\sum_{t} \tilde{V}_{l}(t)V_{k}(t)$, but they are not the same (as seen clearly by the difference between red and green datapoints in Fig. \ref{fig_eeg_art_reject}D. This relationship is addressed by a balanced downsampling so that trial-pairs of projections are only included once (as described above)."

When testing the calibration of the statistics, it is important to be clear about the assumptions about the nature of the noise on each trial. Are there assumptions about (i) the distributional form of the noise, (ii) the independence of noise samples across time-points and (iii) the consistency of noise-variance across trials? Generally, we expect that the performance of the method may be sensitive to violations of some of these assumptions, and robust to violations of others, but which are which? It should be possible to explore some of these points in simulation, as in Figure 7.

We have explored these points in simulation as suggested, as illustrated in figures R5, R6, and R7. These show that there are not assumptions about the distributional form of the noise, the independence of noise samples across time-points (except for the shorter C(t) that may come with correlated brown noise and very low signal), nor the consistency of noise-variance across trials.

* 3 * Regarding the method for determining the duration of the response, the tests in Figure 7A-C are a simple and beauitful self-consistency demonstration for the proposed method. However, does this self-consistency effect persist in the presence of noise? If not, it would be helpful to give the reader some sense of which kinds of noise levels are most likely to lead the method to lose this self-consistency.

The persistence of self-consistency in the presence of noise from the shape in 7(A) is illustrated in 7(D-E-F), and it is also illustrated in response figure R5 above.

Is there a way for the end-user to know what level of noise they are dealing with in empirical data?

The end-user is able to quantify "noise" by the residual term $\sqrt{\varepsilon^T \varepsilon}$ which carries units μV • $\sqrt{\#samples}$. This noise term includes true noise (from amplifiers, environment, etc) as well as ongoing brain activity that is reflected in voltage traces that aren't time-locked to stimulation.

$\sqrt{\varepsilon^T \varepsilon}$ can be divided by $\sqrt{\#samples}$ to obtain a scalar single trial summary term quantifying the magnitude of the residual in microvolts.

This "noise" can also be quantified relative to the coefficient alpha by SNR, or to the total signal variance by explained variance.

This is described in the second-to-last paragraph of the section "Parameterization of single trials by canonical CRP shapes, $C(t)$, magnitude of the voltage response, α_{k}', and the residual, ε_k".

"The residual term, $\varepsilon_{k}(t)$, is a signal that reflects all local brain activity not directly linked to stimulation timing, combined with measurement noise (i.e. from amplifiers and the environment). For example, if a researcher wishes to examine non-phase-locked oscillatory (rhythmic) activity resulting from the stimulation, they should calculate this from $\varepsilon_{k}(t)$ rather than $V_{k}(t)$, since the shape of the deflections of the evoked potential $C(t)$ will have corresponding power in the Fourier domain. For example, a positive deflection in a component of $C(t)$ lasting 100ms will have power at $1/(2 \cdot 0.1 s)=$ 5Hz, but not be an oscillation. Extracts of broadband spectral activity (spread across all frequencies according to a power-law form, but often captured at high frequency by researchers) that capture local brain activity \cite{miller2009power} might be best extracted from $\varepsilon_{k}(t)$ rather than $V_{k}(t)$."

* 4 * When normalizing the single-trial cross-projections (Figure 8), the authors emphasize that a semi-normalized approach is best. In this approach, we compute the dot product between one unit-normalized vector and another unnormalized vector. I wonder if there is an alternative that is superior: instead of normalizing by the length of the V_k vector (|V_k|), normalize by the geometric mean of the lengths of the V_k vector and the V_l vector (sqrt(|V_k||V_l|)?

This a good suggestion. We implement it by modifying the code of the subfunction "ccep_proj" from the function "CRP_method" is changed to:

```
V0=V./(ones(size(V,1),1)*((sum(V.^2,1).^.5).^.5));
```

and

```
P=V0.'*V0;
```

We then apply the CRP method to the first 4 sets from figure 5, we find that the results are essentially identical.

- -

input file: Fig5_sampledata_1
mean projection (S) at response duration: 225.693
response duration, \tau_R: 0.54443
mean alpha-prime: 322.8282
mean SNR: 4.5566
mean explained variance: 0.94851
- -
input file: Fig5_sampledata_2
mean projection (S) at response duration: 18.1856
response duration, \tau_R: 0.24902
mean alpha-prime: 49.1311
mean SNR: 1.6313
mean explained variance: 0.65714
- -
input file: Fig5_sampledata_3
mean projection (S) at response duration: 11.2676
response duration, \tau_R: 0.20996
mean alpha-prime: 38.8708
mean SNR: 1.6491
mean explained variance: 0.56228
- -
input file: Fig5_sampledata_4
mean projection (S) at response duration: 12.6088
response duration, \tau_R: 0.20264
mean alpha-prime: 31.8558
mean SNR: 2.8499
mean explained variance: 0.86286
- -

The advantage of this approach, as far as I can tell, is that it would give less weight to outlier trials, and would be less susceptible to the influence of individual low-amplitude trials, because it combines information from both the row and the column in the P(k,l) matrix.

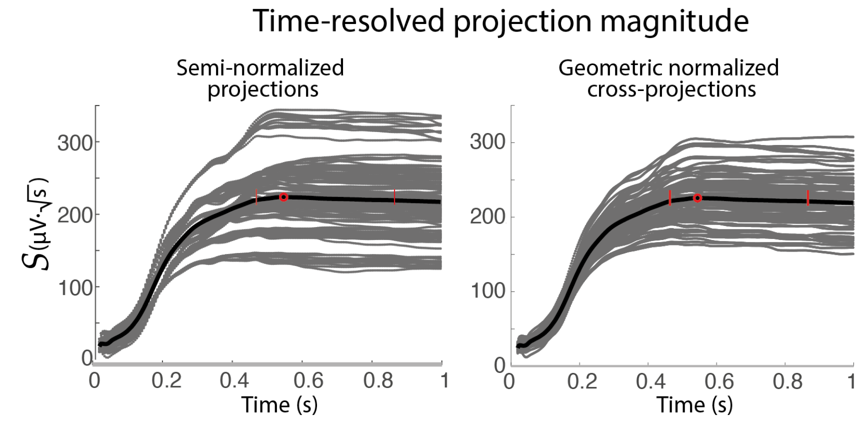

Figure R8: Time-resolved projection magnitudes using the semi-normalized approach (left) and the proposed geometric mean approach (right). The example of this is from the set in the top row of figure 5.

Figure R8 above shows that the distribution of projections is tighter if this “geometric mean” normalization is done. This has higher statistical significance, but will also de-emphasize anomalous trials (i.e. those that are most representative, or those that are most likely to be artifactual). This is something that we very much would like to preserve. However, we do note in a new bullet point in the “future exploration” list at the end of the manuscript:

“\item It is possible to calculate cross-projections using an alternative approach: Instead of normalizing one trial by its norm, and not normalizing the other, one can normalize both trials by the square root of their respective norms. This has the effect of tightening the distribution of projections - generating higher extraction significance, but also de-emphasizing anomalous trials (i.e. those that are most representative, or those that are most likely to be artifactual).”

* 5 * Line 467: “ In the first of these (Figs. 6C and D), a seemingly insignificant response has a very significant brief structure at the beginning of the examined period. This situation may arise when a latent effect of stimulation artifact “carries forward” into the window being considered. Determining what is stimulation artifact and what is brief evoked neural activity is a nuanced topic that we defer to future study.”

Since cortico-cortical axonal conduction latencies are typically 10ms or longer, why not exclude (by default) the first 10ms post-stimulation from the process used to extract C(t) and measure the response duration? This may not fully address the issue, but it should reduce its influence?

Thank you for the recommendation. We do exclude the first 15ms. For the illustrations in this manuscript, we chose t_1=15ms and t_2=1s.

t_1 of 15ms was chosen to reduce the likelihood of contamination by stimulation artifact, and t_2 of 1s was chosen because (anecdotally) the vast majority of sEEG CCEPs we have observed return to baseline well before then.

We anticipate that researchers will adjust t_1 and t_2 based upon their own circumstances.

We note this in text added to the “Data structure” section of the methods:

“In this manuscript, t_1 of 15ms was chosen to reduce the likelihood of contamination by stimulation artifact, and t_2 of 1s was chosen because (anecdotally) the vast majority of sEEG CCEPs we have observed return to baseline well before then. We anticipate that researchers will adjust t_1 and t_2 based upon their own circumstances.”

* 6 * Line 472: “The second artifactual condition to consider (Figs. 6E and F) is the case where a set of responses appear to be complete noise, but the time-resolved projection magnitude $\bar{S}t$ grows steadily in time. Inappropriate baselining of the data produces this - we also defer comprehensive exploration of this to future treatment.”
I understand that there is not space for a full treatment of this point, but it seems important to add a couple of sentences here, elaborating on how inappropriate baselining can be avoided and / or how the end-user can detect when the baseline is incorrect.

Thank you for the suggestion. We have added text to this effect in the “Data structure” section of the Materials and methods:

“Before data are analyzed and parameterized, one should evaluate the preprocessed data for baseline fidelity. Issues with inappropriate referencing or lack of a baseline around zero can be avoided by visual inspection or calculation of the mean and/or median values at far from stimulation times.”

Furthermore, it seems important to emphasize the importance of baselining for the success of this method. In many dimensionality reduction methods, the results are invariant to the centering approach (i.e. you get the same results if you z-score the data and if you don’t), but with this method, it appears that the absolute magnitude and sign of the voltage time-courses are being (successfully) exploited to determine which trials are more important and contain more

signal. Maybe it would be worth emphasizing, around line 93, that the data are not zero-centered on each trial, but they should instead be centered relative to an independently-measured baseline reference.

Thank you for the suggestion. We have added text following the sentence "We define this peak time as the "response duration", or τR.", in the "Response Duration" section of the methods:

"It is important to note that this CRP approach is very sensitive to baselining, as the sign of the voltage in cross-projections around zero determines when the profile of $\Bar{S}_{t}$ begins to decrease."

* 7 * The Introduction of the manuscript would be more compelling if it stated, earlier on, the scientific gap or need that is filled by the present work. It would be nice if the manuscript stated very early on, both: (i) the shortcomings of current approaches, and (ii) the top reason(s) why this new method is important and useful. There is plenty of text making these points in the manuscript, but this motivational info is spread across the Intro, Results, and Discussion.

Thank you for the suggestion. We have added the following to the end of the first paragraph of the introduction:

"Contemporary analysis of these CCEP responses has suffered from reliance on pre-defined assumptions about the shape that the response should have and quantification of effect only by the voltage at a particular time. This manuscript describes an algorithmic approach to formalize and simplify CCEP analysis so that responses of different shape, duration, and magnitude may be quantitatively compared with one another."

Typos / Minor Points

** The method proposed here assumes that there is a single consistent response (the C(t)) which we wish to extract. Are there conditions under which it would make sense to extract multiple responses, i.e. additional columns of the matrix X, line 139, and would these additional responses need to be orthogonal?)

Thank you for the suggestion. We have added a new bullet point addressing this in the list portion of the subsection "Limitations, alternative considerations, and future technical strategies" of the discussion:

"It may be useful in future studies to keep additional columns of \textbf{X} in the linear kernel PCA to study variation across trials (i.e. the second-order moment in the data), rather than the first column alone which is our canonical CCEP shape $C(t)$ (a robust approximation of the mean)."

Note that these would need to be orthogonal to serve such a role.

Line 18: "To address this, we recently introduced a set of four basic paradigms for interpreting CCEP data" — I think that it would be more accurate to say that terminology was recently introduced, or a conceptual framework was introduced, as these paradigms are many years old?

The reviewer is correct. We have changed the text to read:

"To address this, we recently introduced *a conceptual framework formalizing* four basic paradigms…"

Line 36: "stimulated-at" can be "stimulated"

Thank you, we have made the change as suggested.

Line 107: "Because S^bar may be thought of as a measure of mutual information between responses…" — Please justify or elaborate on this point.

\Bar{S} quantifies the correlated deviations from 0V. If we believe that non-zero voltage reflects information in the signal, then the S would reflect mutual information: Put differently, the un-normalized timeseries V_l carries the magnitude, and projecting the normalized shape \tilde{V}_k quantifies the shared shape. We have changed the text to read as follows:

"…may be thought of as a *reflection* of mutual information between responses *(i.e. their correlated deviation from 0V)*, …"

Tiny p-values: In some place, e.g. in the caption of Figure 5, the manuscript reports p-values of the form p << 10^{-34}, but such numbers are so vast (or tiny) that they are almost meaningless. I am not sure of the best way to handle this, but maybe something like "p approximately 0", or "p-value negligible" or "p << 0.001" is better?

This is a good recommendation, and we agree that it would improve readability of the manuscript. However, the current trend in publishing has been to publish precise p-values. With that in mind, we discussed whether to go with the approximate statements or precise p-values. Our consensus was to proceed with precise p-values.

Figure 6A: What is "extraction significance" in the title?

We initially defined extraction significance in the methods subsection "Quantifying significance and identifying anomalous single trials" as "The set of projection magnitudes $S_{\tau R}$ can be tested against zero for significance, which we call the "extraction significance"."

To make this clearer, we have modified the methods subsection title to "Extraction Significance: Quantifying reliability of response structure and identifying anomalous single trials".

Line 365: "How the recorded C(t) curves isolated in these regions of the brain differ from one another, if similar input types/regions can be approximated, might serve as an informative model to understand how intralaminar dynamics result in characteristic voltage responses."
… Perhaps this would read better as: "Therefore, consistent differences in C(t) across these regions could inform new models of how intralaminar dynamics generate characteristic voltage responses."

Thank you, we have made the change as suggested.
